# A randomised controlled trial confirms the non‐superiority of bone marrow aspirate (BMA) from the posterior iliac crest and proximal tibia compared to platelet rich plasma (PRP) in the treatment of knee osteoarthritis

**DOI:** 10.1002/jeo2.70442

**Published:** 2025-10-09

**Authors:** Elisabetta Mormone, Leonardo Savastano, Valeria Guerra, Marco Sandri, Francesco Maruccia, Giovanni Rossi, Gennaro Di Maggio, Nicola Pio Sinisi, Franco Lucio Gorgoglione

**Affiliations:** ^1^ Institute for Stem Cell Biology, Regenerative Medicine and Innovative Therapies (ISBReMIT) Fondazione IRCCS “Casa Sollievo della Sofferenza” San Giovanni Rotondo Foggia Italy; ^2^ Department of Orthopedics and Trauma Surgery Fondazione IRCCS “Casa Sollievo della Sofferenza” San Giovanni Rotondo Foggia Italy; ^3^ Big and Open Data Innovation Laboratory (BODaI‐Lab) University of Brescia Brescia Italy; ^4^ Department of Hematology, Stem Cell Transplant Unit Fondazione IRCCS Casa Sollievo della Sofferenza San Giovanni Rotondo Foggia Italy

**Keywords:** BMA, knee osteoarthritis (KOA), Marrow Cellution™ Aspiration System, MSCs, PRP

## Abstract

**Purpose:**

The reported clinical trial aimed to determine the clinical efficacy of ex vivo mesenchymal stromal cells (MSCs) derived from bone marrow (BM), aspirated from the posterior iliac crest and proximal tibia, in comparison to autologous platelet‐rich plasma (PRP) for the knee osteoarthritis (KOA) treatment and to assess the differences in cellular yield between the two harvest sites.

**Methods:**

A single‐centre, parallel, randomised controlled trial was designed to investigate the clinical effects of bone marrow aspirate (BMA) from the posterior iliac crest compared to BMA from the proximal tibia in treating KOA, with a control group receiving PRP. Ninety patients with KOA were divided equally among the three groups. Visual Analogue Score (VAS) and Western Ontario and McMaster Universities Arthritis Index (WOMAC) score were used for clinical outcome evaluation at 6 months after the treatment, and cellular analysis of BMA was performed.

**Results:**

Cell count confirmed that the posterior iliac crest was significantly more densely populated with mononuclear cells than the proximal tibia. Flow cytometric analysis of ex vivo BMA also confirmed a significantly greater number of MSCs in the BM‐derived from the posterior iliac crest when compared with the proximal tibia, together with a significantly higher number of platelets. The analysis confirmed that the improvement in early pain and function scores after each treatment was statistically significant within each of the three groups (median VAS decrease: PRP Group −2 (*p* < 0.001), Crest Group −2 (*p* < 0.001), Tibia Group −2 (*p* < 0.001)), (median WOMAC decrease: PRP Group −13.5 (*p* < 0.001), Crest Group −12.5 (*p* < 0.001), Tibia Group −12 (*p* < 0.001)). However, no statistically significant differences were observed in the improvements among the three study groups. A significantly greater improvement in VAS was observed in patients with Kellgren Lawrence (KL) I–II. In the Tibia Group, the effect of BMA on the change in VAS was associated with BMI and %MSCs, whereas in the Crest Group, the change in WOMAC score was associated with %BM and platelet count.

**Conclusion:**

The iliac crest yields a higher concentration of MSCs compared to the proximal tibia. However, both sources demonstrated a beneficial clinical outcome in the treatment of KOA, with no evidence of superiority over PRP treatment.

**Level of Evidence:**

Level I.

**Abbreviations**
BM: bone marrow
BMA: bone marrow aspirate
BMAC: bone marrow aspirate concentrate or concentration
BMI: body mass index
IA: intra‐articular injections
KL: Kellgren–Lawrence
MC: marrow cellution
MNCs: mononuclear cells
MSCs: mesenchymal stromal cell(s)
OA: osteoarthritis
PLTs: platelets
PRP: platelet rich plasma
SD: standard deviation
VAS: visual analogue score
WOMAC: Western Ontario and McMaster Universities Arthritis Index

## INTRODUCTION

Osteoarthritis (OA) is a long‐term degenerative condition primarily characterised by the progressive deterioration of subchondral bone, followed by damage to the articular cartilage and adjacent synovial tissues [18, 32]. OA is a disabling condition with increasing incidence and prevalence in the general population. The most common risk factors associated with OA are aging, genetic predisposition and obesity [19].

The joint most commonly involved in OA is the knee (KOA), which is affected in about 60%–85% of all OA cases, with higher prevalence in women [10, 23].

Since conventional conservative treatments for KOA (anti‐inflammatory drugs, glucosamine, chondroitin sulphate, omega‐3 fatty acids, hyaluronic acid and corticosteroid injections), have shown limited clinical benefit, failing to prevent disease progression or to provide long‐term improvements in joint function and pain [13], orthobiologics are emerging as an effective alternative for the non‐surgical management of KOA. However, the main limitation of these products lies in the variability of their composition and biological activity. Although current evidence supports their role as ‘symptom‐modifying’ agents, there is little to no evidence that they can promote a true tissue regeneration [17, 31]. Platelet rich plasma (PRP) and bone marrow aspirate (BMA) represent two promising regenerative therapies [17]. Human BM is a source of MSCs, growth factors, and cytokines that can aid in anti‐inflammatory and the regeneration of various tissues, including cartilage and bone. While MSCs in bone marrow occupy only a small fraction (0.001%) of nucleated cells, bone marrow derived products for cartilage pathologies, such as degeneration, cartilage defects, and osteoarthritis, have gained considerable recognition in recent years due to its potential benefits, including disease‐modifying ability and regenerative capacity [6, 20]. The present study aims to compare the clinical outcomes of BMA from the posterior iliac crest compared to BMA from the proximal tibia in treating KOA, with a control group receiving PRP, 6‐month post‐treatment, and to characterise the cellular composition of BMA from the two sources.

## METHODS

### Study design

Ninety patients with KOA were enroled in a parallel‐randomised controlled unblinded study and assigned to three groups: 30 patients received BMA from the posterior iliac crest (Crest Group), 30 received BMA from the proximal tibia bone (Tibia Group), and 30 received PRP (PRP Group) as control. Randomisation was performed by the biostatistician using a computer‐generated randomisation sequence. The mean age at the time of treatment was 53.8 ± 7.9 years in the Crest Group, 51.3 ± 9.7 years in the PRP Group, and 52.9 ± 8.8 years in the Tibia Group (Table 2). No significant difference was observed in the gender distribution (*p* = 0.6), indicating that sex was well balanced across the three groups. However, Kellgren–Lawrence (KL) grade was not equally distributed among the groups (*p* = 0.03) (Table 2). Here, we present 6‐month follow‐up results for the 30 patients in each group.

### Study population and main criteria for inclusion/exclusion

Patients were eligible for inclusion if they met all of the following criteria:

− 35 y.o.≤ Age ≤ 65 y.o
− Uni‐compartimental knee osteoarthritis grade I–IV KL
− Failure of conservative treatment with corticosteroids
− Willingness to participate in the study and signed informed consent
− Ability to provide written, personally signed, and dated informed consent to participate in the study, in accordance with the ICH GCP Guideline E6 and applicable regulations, before completing any study‐related procedures
− Sufficient understanding, ability, and willingness to comply fully with study procedures and restrictions

Patients were excluded if they met any of the following conditions:

− History of trauma to the knee, leg or pelvic trauma within the previous 6 months
− Active or prior neoplastic disease
− Rheumatic diseases
− Constitutional deformity of the lower limb > 10°
− Body mass index (BMI) ≤ 18, BMI ≥ 35
− Pregnancy
− Participation as an investigator, sub‐investigator, study coordinator or staff member of the study, or being an immediate family member of any of the aforementioned
− Any condition that, in the opinion of the Investigator, would jeopardise the evaluation or safety or be associated with poor adherence to the protocol.

### Evaluation methods for clinical outcomes

The clinical outcomes of the treatments were evaluated through the examination of the patients at 0 and 6‐month follow‐up, collecting information on the evolution of pain and knee function through dedicated scores Visual Analogue Score (VAS) and Western Ontario and McMaster Universities Arthritis Index (WOMAC). WOMAC subscales for pain, stiffness, and physical function were also analysed.

### PRP preparation

PRP was prepared at the Transfusion Centre of Casa Sollievo della Sofferenza Hospital, using a manual, closed system as follows: 150 mL peripheral blood was collected before the treatment and centrifuged for 15 min at 900 rpm, 22°C. Plasma and buffy coat were collected and platelets (PLTs) were counted. Afterwards they were centrifuged again for 12 min at 3338 rpm. After the centrifugation plasma was removed in order to have a final PLTs concentration of 0.8–1 × 10^6^/μL and the resulting PRP was stored at −30°C.

### BM aspiration technique

**Figure 1:**
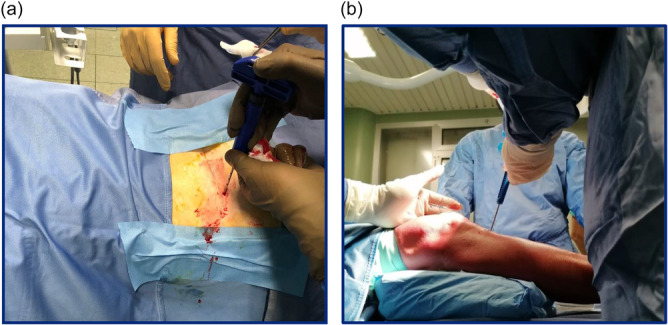
Bone marrow aspiration from posterior iliac crest (a) and proximal tibia (b) using Marrow Cellution™ Bone Marrow Aspiration System.

BM aspiration was performed using the Marrow Cellution™ Bone Marrow Aspiration System (ASPIRE MEDICAL INNOVATION), an advanced device specifically designed to optimise the yield of stromal and progenitor cells in the final aspirate [27]. A total of 10 mL of bone marrow was harvested under mild sedation, either from the posterior iliac crest or the proximal tibia, according to the randomisation assignment. (Figure 1). Five mL of aspirate were injected into the knee of the same patient, and the remaining 5 mL were collected in tubes containing 1000 U/mL of heparin (Sigma‐Aldrich, St. Louis, MO, USA), for the analysis, described below. The procedure was performed in the operating room by multiple surgeons.

### BM purity and mononuclear cells (MNC) count

The day before the BM harvesting, a sample of venous blood was taken for a complete blood count. On the day of the injection, a small amount of withdrawn BM was used for the complete blood count. Cells were counted using an automatic Hematology Analyzer ABX Micros ES 60 (HORIBA Medical). The number of leucocytes and erythrocytes of BM and peripheral blood (PB) was used for calculating the BM purity (admixture of peripheral blood in the bone marrow aspirates) according to Holdrinet et al. [16]: BM purity = [1 − (erythrocytes_BM_/erythrocytes_PB_) × (leucocytes_PB_/leucocytes_BM_)] × 100%. MNCs of BM were derived by the summary of lymphocytes and monocytes count.

### Flow cytometric analysis

Erythrocyte‐lysed whole BM samples were immunophenotyped using an eight‐colour direct immunofluorescence panel technique. The following combination was used to identify MSCs: CD45‐V500/CD19‐V450/CD71‐APC‐H7/CD105‐PerCP‐Cy5.5/CD34‐PE‐Cy7/CD271‐PE/CD73‐FITC/HLADR‐APC. Monoclonal antibodies were purchased from BD Biosciences. Gating strategy to identify MSCs was performed as follows: (1) the CD271‐positive population was selected; (2) the CD271‐positive events which expressed both CD73 and CD105 markers were gated; (3) finally, a back‐gate on CD45‐negative/low expressed events was provided to confirm the population previously defined as MSCs [12]. MSCs were quantified as percentage of total BM cells. An intra‐assay quality check of the whole cell sample was provided by the identification of B‐cell precursors (CD19+, HLADR+, CD45+lo), hematopoietic stem cells (CD34+, HLADR+, CD45+int), and nucleated red blood cells (CD71+, HLADR‐, CD45‐). In all samples, an isotype matched negative control with no BM reactivity was used. At least 100.000 events were acquired by using a FACS Canto flow cytometer and a FACS Diva software.

### PRP and BMA injection

For intra‐articular knee injections with the patient lying down in a supine position, a superolateral injection approach was preferred, especially when an effusion was present. The physician was standing on the injection side of the affected knee and injected 5 mL of PRP, for the PRP treatment, or BMA, for the BMA treatment, through intra‐articular knee injection, using a needle with 27–22 gauge and 1.5–2.0 inches. In presence of a swollen knee, a needle with a gauge of 22–18 and a length of 1.5–2.0 inches was used for preliminary arthrocentesis, before the injection of orthobiologics. PRP treatment was repeated three times every 7 days, while BMA treatment only once. PRP injections were performed on an outpatient basis, whereas the BMA injection was carried out in the operating room. Both treatments were administered by multiple physicians.

### Statistical analysis

A total sample size of 90 patients (30 per group) was calculated to provide at least 95% power, with a two‐sided Type I error rate of 5%, to detect a statistically significant difference in the primary endpoint between the PRP Group (*n* = 30) and the combined bone marrow groups (*n* = 60), using a two‐sided unpaired t‐test. The calculation assumed a mean baseline‐to‐12‐month change in WOMAC score of –10 points (SD = 6) in the PRP Group and –15 points (SD = 6) in the bone marrow groups.

Continuous variables were described using medians and interquartile ranges (IQRs), while categorical variables were reported as absolute and relative frequencies. Differences in continuous variables among the three study groups were evaluated using the Kruskal–Wallis test, a non‐parametric method suitable for comparing multiple independent groups. For categorical variables, Fisher's exact test was applied to assess group differences. To analyse within‐group changes in VAS and WOMAC scores between baseline (T0) and the 6‐month follow‐up (T6), the Wilcoxon matched‐pairs signed‐rank test was used. Relationships between continuous variables were explored through Spearman's rank correlation coefficient, whereas associations between a continuous variable and a categorical variable were quantified using the coefficient of determination (*R*²), which represents the proportion of variance explained. To account for multiple variables simultaneously and adjust for potential confounders, multivariable linear regression models were applied. A *p*‐value below 0.05 was considered to indicate statistical significance. All statistical analyses were conducted using Stata 18 (StataCorp, 2023, College Station, TX).

## RESULTS

### Clinical outcome: BMA versus PRP treatment

**Figure 2:**
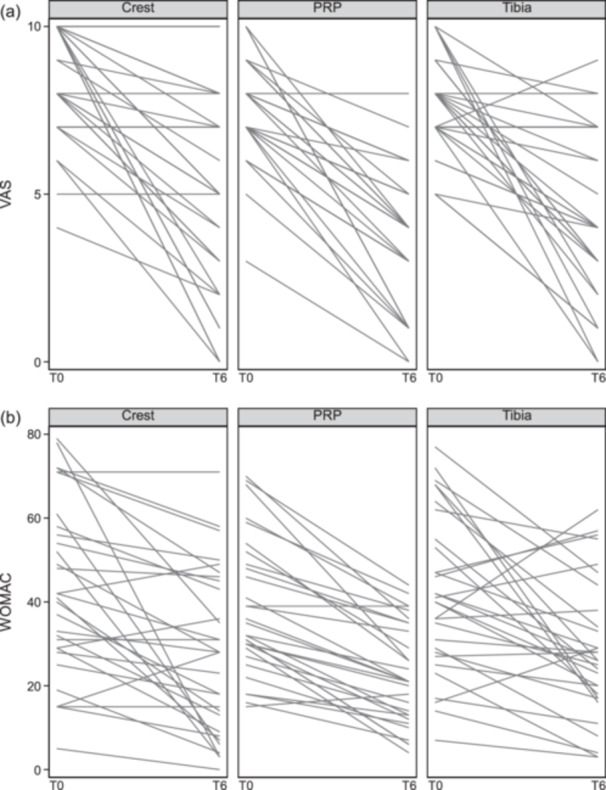
VAS (a) and WOMAC (b) values for each patient in the three study groups before and after treatment. VAS, Visual analogue score; WOMAC, Western Ontario and McMaster Universities Arthritis Index.

Figure 2a indicates that, out of 30 patients, four in the Crest Group and one in the PRP Group showed no improvement in VAS, while in the Tibia Group, one patient worsened slightly and two showed no improvement. Figure 2b indicates that in the Crest Group, three patients worsened and three showed no improvement in WOMAC; in the PRP Group, two patients slightly worsened; and in the Tibia Group, seven patients worsened. Statistical analysis evidenced significant clinical efficacy for both BMA and PRP treatments, as indicated by changes in VAS and WOMAC scores before and after treatment with median values and IQRs reported in Table 1 and means with standard deviations shown in Supporting Information: Table S1 (Table 1 and Supporting Information: Table S1). Median VAS decreases were −3 for the PRP Group (*p* < 0.001), −3 for the Crest Group (*p* < 0.001) and −3 for the Tibia Group (*p* < 0.001). Median WOMAC decreases were −13.5 for the PRP Group (*p* < 0.001), −12.5 for the Crest Group (*p* < 0.001) and −12 for the Tibia Group (*p* < 0.001). The reductions in VAS and WOMAC scores across the three study groups were not significantly different as shown in Table 2 (median, IQR) and Supporting Information: Table S2 (mean ± SD) (Table 2 and Supporting Information: Table S2).

**Table 1:** Median values and interquartile ranges (IQRs) of 6‐month changes in VAS and WOMAC scores across the three study groups.

Variable	Crest	*p* value	PRP	*p* value	Tibia	*p* value
ΔVAS	−3 (−5 to −1)	<0.001	−3 (−5 to −2)	<0.001	−3 (−5 to −1)	<0.001
ΔWOMAC	−12.5 (−21 to −5)	<0.001	−13.5 (−23 to −9)	<0.001	−12 (−25 to −3)	<0.001
ΔWOMAC PAIN	−2 (−5 to 0)	<0.001	−3 (−5 to −2)	<0.001	−2.5 (−5 to 0)	0.001
ΔWOMAC STIF	−1 (−3 to 0)	<0.001	−2 (−3 to −1)	<0.001	−1 (−3 to 1)	0.033
ΔWOMAC PF	−9 (−16 to −3)	<0.001	−9 (−15 to −5)	<0.001	−8 (−21 to −2)	<0.001

Abbreviations: *p* value from the Wilcoxon matched‐pairs test assessing the significance of differences between pre‐ and post‐treatment within each arm; PF, Physical Function; VAS, Visual Analogue Scale; WOMAC, Western Ontario and McMaster Universities Arthritis Index; Δ, 6‐month change, calculated as post‐treatment minus baseline value.

**Table 2:** Summary of patient demographics, baseline clinical characteristics, bone marrow cellular and biochemical composition, and clinical outcome measures, presented separately for each of the three study groups.

	Study groups			
Variable	Crest	PRP	Tibia	*p* value
*N*	30 (33.3%)	30 (33.3%)	30 (33.3%)	
Demographic and baseline clinical characteristics				
Sex female	17 (56.7%)	15 (50.0%)	13 (43.3%)	0.587
Age (years)	56.5 (50–59)	52 (46–58)	54 (46–60)	0.596
BMI (Kg/m^2^)	26.3 (24.8–29.4)	27.5 (26–28.4)	26.9 (24.–8.1)	0.578
Side R	19 (63.3%)	18 (60.0%)	14 (46.7%)	0.387
Kellgren–Lawrence Grading Scale				
I–II	18 (60.0%)	25 (83.3%)	16 (53.3%)	0.037
III–IV	12 (40.0%)	5 (16.7%)	14 (46.7%)	
HKA	0 (−1.3 to 0.7)	−0.3 (−1.6 to 1.6)	0.4 (−0.8 to 1.5)	0.610
Bone marrow cellular and biochemical composition				
%BM	80.5 (71–88)	–	41 (30.5–52)	<0.001
MSCs (10^3^ * %)	80 (38–235)	–	0 (0–4)	<0.001
MNCs (10^6^/mL)	11.3 (5.9–14.4)	–	5.6 (3.5–6.8)	<0.001
Monocytes (10^6^/mL)	2.1 (1.4–3.7)	–	1.1 (0.9–1.8)	0.002
Platelets (10^6^/mL)	237 (188–312)	–	144 (76–234)	0.004
%HCT	39 (36.1–42.5)	–	39.7 (35.2–43.8)	0.855
Clinical outcome measures				
VAS at T0	8.5 (7–10)	7 (7–8)	8 (7–8)	0.028
VAS at T6	5 (2–7)	4 (3–5)	4 (3–7)	0.324
ΔVAS	−3 (−5 to −1)	−3 (−5 to −2)	−3 (−5 to −1)	0.762
WOMAC at T0	41.5 (29–58)	33.5 (29–49)	40.5 (28–55)	0.560
WOMAC at T6	28 (9–45)	21 (13–35)	26 (17–38)	0.514
ΔWOMAC	−12.5 (−21 to −5)	−13.5 (−23 to −9)	−12 (−25 to −3)	0.635
WOMAC Pain at T0	8 (6–12)	7.50 (5–11)	8 (6–11)	0.773
WOMAC Pain at T6	5 (2–7)	5 (2–6)	5 (4–7)	0.381
ΔWOMAC Pain	−2 (−5 to 0)	−3 (−5 to −2)	−2.50 (−5 to 0)	0.311
WOMAC Stiffness at T0	4 (2–6)	4 (2–5)	4 (2–6)	0.834
WOMAC Stiffness at T6	2 (1–3)	2 (1–3)	2 (1–3)	0.637
ΔWOMAC Stiffness	−1 (−3 to 0)	−2 (−3 to −1)	−1 (−3 to 1)	0.148
WOMAC PF at T0	28.5 (21–43)	22 (19–34)	30 (20–39)	0.427
WOMAC PF at T6	19 (7–34)	14 (8–26)	18 (12–32)	0.529
ΔWOMAC PF	−9 (−16 to −3)	−9 (−15 to −5)	−8 (−21 to −2)	0.871

*Note*: Medians with interquartile ranges summarised continuous variables, while frequencies and percentages described categorical variables.

Abbreviations: Δ, absolute variation between T6 and T0 (T6 − T0); BMI, body mass index; %HCT, haematocrit percentage; HKA, hip‐knee‐ankle angle; MNCs, mononuclear cells; PF, physical function; %BM, percentage of bone marrow contribution; %MSCs, percentage mesenchymal stromal cells; T0, baseline; T6, 6‐month timepoint; VAS, visual analogue scale; WOMAC, Western Ontario and McMaster Universities osteoarthritis index.

### PRP and BMA treatment

The technique used to obtain BM samples from the proximal tibia and posterior iliac crest was straightforward and reproducible in all 60 patients. No complications, such as fractures or neurovascular damage, occurred in the posterior iliac Crest Group, where only two cases of edema and one haematoma were observed; in the proximal Tibia Group, fractures occurred in two out of 30 patients (6.7%) (*p* < 0.001).

### Bone marrow purity and MNCs concentration

A total of 60 BMA samples were analysed, with 30 samples from Crest Group and 30 from Tibia Group. As expected, BM collected from the proximal tibia was significantly less pure than that from the posterior iliac crest. Specifically, the mean BM purity was 80.5% in the posterior iliac crest and 41% in the proximal tibia (*p* < 0.001) (Table 2 and Supporting Information: Table S2). The difference in BM purity was also reflected in the variation in MNC concentration between the two harvest sites. The mean MNC concentration (in millions of cells per mL) was 11.3 in the posterior iliac crest and 5.6 in the proximal tibia (*p* < 0.001) (Table 2 and Supporting Information: Table S2).

### Quantification of MSCs from the posterior iliac crest and from the proximal tibia

Cellular quantification by flow cytometry showed that the number of MSCs in the BM was significantly higher in the posterior iliac crest (*p* < 0.001). Specifically, MSC levels were ≤ 0.080% in at least 50% of the samples from the Crest Group, whereas more than 50% of samples from the Tibia Group had no detectable MSCs (Table 2 and Supporting Information: Table S2).

### Association between VAS change and secondary parameters

**Figure 3:**
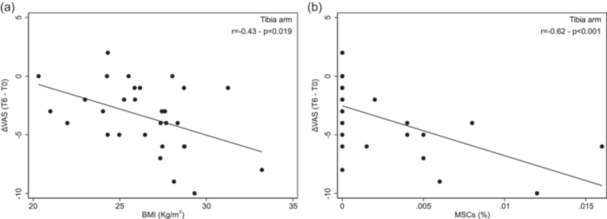
Association between pre/post **VAS** change and BMI (a) in the Tibia Group. Association between pre/post **VAS** change and %MSCs (b) in the Tibia Group. BMI, body mass index; %MSCs, percentage mesenchymal stromal cells; VAS, visual analogue scale.

Data in Table 3 show a significant negative correlation between the pre/post change in VAS and BMI only in the Tibia Group (*r *= −0.43, *p* = 0.019) (Figure 3a). Additionally, an analysis of the correlation between the change in VAS and %MSCs indicates that a higher %MSCs is associated with a greater effect of BMA treatment (*r* = −0.62, *p* < 0.001) (Table 3 and Figure 3b). In the Crest and PRP Groups, VAS change differed significantly across KL grades, with patients classified as KL I–II showing a better response than those with KL III–IV (Table 3 **)**. Multivariable regression analysis confirmed that the association between pre/post VAS change and BMI and %MSCs was present only in the Tibia Group and also confirmed the significant association between VAS change and KL grade.

**Table 3:** Association between pre/post changes in VAS and clinical variables within the three groups: Spearman's rank correlation for pairs of continuous variables, and the coefficient of determination (*R*²) for associations between a continuous and a categorical variable.

Variable	Crest	*p* value	PRP	*p* value	Tibia	*p* value
Age	0.04	0.827	0.12	0.538	−0.33	0.075
BMI	−0.18	0.341	−0.05	0.809	−0.43	0.019
HKA	0.13	0.497	−0.20	0.296	0.17	0.355
%HCT	−0.17	0.389	–	–	0.09	0.665
%BM	−0.13	0.528	–	–	−0.02	0.944
%MSCs	0.26	0.169	–	–	−0.62	0.000
MNCs	0.16	0.434	–	–	0.20	0.332
Monocytes	0.07	0.711	–	–	0.28	0.179
PLT	0.16	0.413	–	–	0.16	0.441
Sex	0.08	0.151	0.01	0.554	0.01	0.687
Side	0.02	0.522	0.00	0.971	0.24	0.006
KL I + II vs III + IV	0.32	0.000	0.18	0.001	0.00	0.994

Abbreviations: BMI, body mass index; %HCT, haematocrit percentage; HKA, hip‐knee‐ankle angle; KL, Kellgren–Lawrence scale; MNCs, mononuclear cells; %BM, percentage of bone marrow contribution; %MSCs, percentage mesenchymal stromal cells; PLT, platelet; VAS, visual analogue scale.

### Association between WOMAC change and secondary parameters

**Figure 4:**
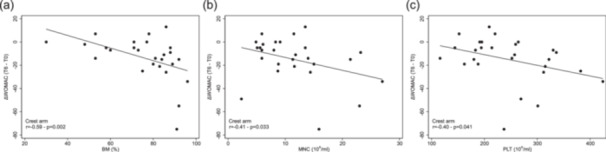
Association between pre/post **WOMAC** change and (a) %BM in the Crest Group, (b) MNC count in the Crest Group, and (c) PLT count in the Tibia Group. MNCs, mononuclear cells; %BM, percentage of bone marrow contribution; WOMAC, Western Ontario and McMaster Universities osteoarthritis index.

A significant correlation between the pre/post change in WOMAC score and %BM was found, but only in the Crest Group (*r* = −0.59, *p* = 0.02) (Table 4 and Figure 4a). This indicates that increasing the purity of BM withdrawal enhances the clinical effect of the BMA treatment, when BM is derived from the iliac crest. Moreover, in the same group, correlations were also observed between the pre/post change in WOMAC score and MNCs (*r* = −0.41, *p* = 0.03), and between the pre/post change in WOMAC score and PLT (*r* =−0.40, *p* = 0.041) (Table 4 and Figure 4b,c). On the contrary, no significant correlation was found between the pre/post change in WOMAC score and the number of monocytes in the Tibia Group, as observed in the intermediate study. Multivariable regression analysis confirmed the association between WOMAC score change and %BM, as well as between WOMAC score change and PLT when BMA is derived from the iliac crest.

**Table 4:** Association between pre/post changes in WOMAC and clinical variables within the three groups: Spearman's rank correlation for pairs of continuous variables, and the coefficient of determination (*R*²) for associations between a continuous and a categorical variable.

Variable	Crest	*p* value	PRP	*p* value	Tibia	*p* value
Age	0.15	0.417	−0.07	0.720	−0.41	0.024
BMI	−0.22	0.234	−0.22	0.244	−0.30	0.113
HKA	−0.18	0.331	0.08	0.679	−0.01	0.943
%HCT	0.07	0.744	–	–	−0.37	0.066
%BM	−0.59	0.002	–	–	0.19	0.373
%MSCs	0.05	0.778	–	–	−0.22	0.232
MNCs	−0.41	0.033	–	–	−0.03	0.884
Monocytes	−0.30	0.123	–	–	0.08	0.694
PLT	−0.40	0.041	–	–	0.13	0.541
Sex	0.06	0.154	0.00	0.843	0.05	0.259
Side	0.06	0.159	0.09	0.115	0.02	0.468
KL I + II vs III + IV	0.13	0.034	0.02	0.427	0.00	0.896

Abbreviations: BMI, body mass index; %HCT, haematocrit percentage; HKA, hip‐knee‐ankle angle; MNCs, mononuclear cells; %BM, percentage of bone marrow contribution; %MSCs, percentage of mesenchymal stromal cells; PLT, platelet; KL, Kellgren–Lawrence scale; WOMAC, Western Ontario and McMaster Universities osteoarthritis index.

## DISCUSSION

The main objective of this study was to evaluate the clinical effects of BMA obtained from the proximal tibia and posterior iliac crest, in terms of functional improvement (WOMAC score) and pain reduction (VAS score), at 6‐months from the treatment, in relation to their ex vivo MSCs composition, compared to PRP.

The rationale for this comparison is based on the growing clinical use of orthobiologic therapies and the current lack of consensus regarding the optimal source and preparation of bone marrow‐derived treatments.

As reported in other works, MSCs concentration varies across different anatomical sites and depends on the harvesting device and technique used [7, 8, 9, 24, 33]. In this study we used PRP as an autologous source of healing factors, as the control, because previous clinical studies have shown the superior clinical outcome compared to other conventional injectable treatments for symptomatic KOA [22, 30].

First, data confirmed that the BM purity is higher in the BM aspirated from the posterior iliac crest than that aspirated from the proximal tibia, with values exceeding those reported in the intermediate analysis (80.5% vs. 71% for the crest; 41% vs. 30% for the tibia).

As also reported in other studies, the posterior iliac crest was significantly more densely populated with MNCs than the proximal tibia [9, 25, 26], with a 2:1 ratio (11.3 × 10^6^/mL and 5.6 × 10^6^/mL) consistent with the intermediate analysis. However, unlike previous findings, monocyte levels were not comparable between the two sites. In the present study, monocyte levels in the iliac crest were noticeably higher than those observed in the intermediate analysis (2.1 × 10⁶/mL vs. 1.55 × 10⁶/mL) and lower in the proximal tibia (1.1 × 10⁶/mL vs. 1.53 × 10⁶/mL).

Moreover, a significantly higher percentage of *ex vivo* MSCs was found in BM derived from the posterior iliac crest compared to the proximal tibia, within the range for MSCs in the whole bone marrow (0.01%–0.1%) [1, 14]. Specifically, %MSCs evaluation in the posterior iliac crest showed a moderately higher concentration (0.08%) compared to the previous intermediate analysis (0.05%). In contrast, MSCs were undetectable in most samples from the proximal tibia. These findings support the posterior iliac crest as the gold standard site for BMA harvesting, widely used in regenerative therapies for KOA [25]. Furthermore, no differences were observed in phenotype, as demonstrated by flow cytometric analysis, and in morphology, as observed by MSCs expanded in vitro for up to 8 weeks (data not shown).

The analysis in the present study confirmed that BMA from the posterior iliac crest contained a significantly higher number of PLTs compared to the proximal tibia (237 × 10^6^/mL and 144 × 10^6^/mL, *p* < 0.002). However, the PLT count in the proximal tibia was double compared to that calculated on 15 patients, as shown previously. Moreover, the PLT count in the posterior iliac crest exceeded that reported by others using the same device and harvesting the same BM volume [7]. Although BMA from the posterior iliac crest exhibited greater purity, a higher %MSCs, and a larger number of PLTs, clinical data from the present study showed no significant differences in ΔWOMAC and ΔVAS across the three groups 6‐months after treatment, consistent with the intermediate analysis [24]. These findings align also with other clinical studies that have not demonstrated the superiority of BMAC over PRP [2, 5, 11, 28]. We found that the improvement in early pain and function scores after treatment was statistically significant in all three groups, although slightly lower than the values observed in the intermediate analysis. Moreover, the highest ΔWOMAC was observed in PRP Group (−13.5) rather than in posterior iliac crest as found previously, although no significant differences were found among the three groups.

Conversely, ΔVAS showed similar results across all three groups. The effect of ΔVAS was influenced by KL grade when considering BMA from the posterior iliac crest and PRP, with patients classified as KL I–II exhibiting better outcomes, consistent with previous findings. These results were confirmed by multivariable regression analysis. Recently, other studies have highlighted the importance of patient selection based on KL grade in the treatment of OA with BMAC [29] and PRP [21]. Moreover, univariable and multivariable regression analyses confirmed a positive correlation between ΔVAS and %MSCs in patients treated with BMA from the Tibia Group, indicating that higher %MSC levels were associated with greater improvements in VAS scores.

Sex had a weak but statistically significant impact on ΔVAS in this study, a finding not previously observed **(**Table 3).

At 6 months, WOMAC analysis showed a correlation between functional improvement and %BM, consistent with previous findings, as well as a correlation with MNCs and PLT, but only in the posterior iliac Crest Group. Multivariable regression analysis confirmed the associations with %BM and PLT, which had not been previously observed.

In contrast, the previously reported correlation between monocytes and ΔWOMAC in the Tibia Group was not replicated in the full sample analysis. Furthermore, in patients treated with BMA, no correlation was observed between MSC count and functional change (ΔWOMAC), confirming the findings from the intermediate analysis [24].

The main finding of this study, namely the lack of clinical superiority of BMA over PRP and the clinical equivalence between the two withdrawal sites, has also been recently confirmed by others [3, 4, 15]. These findings suggest that, in terms of pain relief, BMA and PRP may be considered interchangeable therapeutic options. However, further investigation is needed to determine whether any differences exist between the two treatments in terms of cartilage regeneration.

### Limitations

Although this randomised trial was designed to minimise potential sources of bias, certain intrinsic factors (such as bone quality and comorbidities) could not be fully controlled and may have influenced MSCs yield. While these variables are inherently difficult to standardise, large‐scale, multicenter trials involving more diverse populations and larger sample sizes could enable more robust stratification and statistical adjustments, thereby improving the interpretability and generalisability of the results. In addition, a more refined stratification of patients (based on age, KL grade and comorbidities) could help isolate the effects of these variables on MSCs yield and clinical outcomes. Finally, the inclusion of a placebo group and the use of a blinded study design could further strengthen the evaluation of the true efficacy of these treatments in the management of KOA.

## CONCLUSION AND PERSPECTIVES

Despite significant differences in cellular composition, bone marrow harvested from the posterior iliac crest and proximal tibia showed clinical effects (in terms of VAS and WOMAC) comparable to PRP in patients with OA. This was particularly evident in patients classified as KL I–II, who experienced a significant reduction in pain. As a next step we are planning to investigate whether the observed clinical improvements are associated with cartilage regeneration, as assessed by MRI evaluation.

## AUTHOR CONTRIBUTIONS

Elisabetta Mormone made substantial contributions to the study design, analysis, interpretation of data, in vitro cells culture and construction of the manuscript. Leonardo Savastano and Francesco Maruccia made substantial contributions to the design, collection of data and analysis and interpretation of results. Valeria Guerra contributed to collection of data, analysis and interpretation of results, construction of manuscript. Marco Sandri contributed to the statistical analysis, interpretation of data and manuscript reviewing. Gennaro Di Maggio contributed to collection of data. Giovanni Rossi and Nicola Pio Sinisi contributed substantially to the FACS analysis. Franco Lucio Gorgoglione supervised the entire work. All authors agree to be accountable for all aspects of this manuscript. All authors read and approved the final manuscript.

## CONFLICT OF INTEREST STATEMENT

The authors declare no conflicts of interest.

## ETHICS STATEMENT

The study was approved by the local ethics committee (protocol number 263/01/DG). All subjects gave their written informed consent before procedure.

## Supporting information


**Supplemental Fig** Boxplots for VAS (a) and WOMAC (b) at T0 and T6 in the Crest, PRP, and Tibia Groups, respectively.

Table S1 new.

Table S2.

## Data Availability

The data sets during and/or analysed during the current study are available from the corresponding author on reasonable request.
