# Supplementary figures and images for: A randomised controlled trial confirms the non‐superiority of bone marrow aspirate (BMA) from the posterior iliac crest and proximal tibia compared to platelet rich plasma (PRP) in the treatment of knee osteoarthritis
Source: J Exp Orthop. 2025 Oct 9;12(4):e70442. doi: 10.1002/jeo2.70442 (PMC12509241; doi:10.1002/jeo2.70442)

**a**

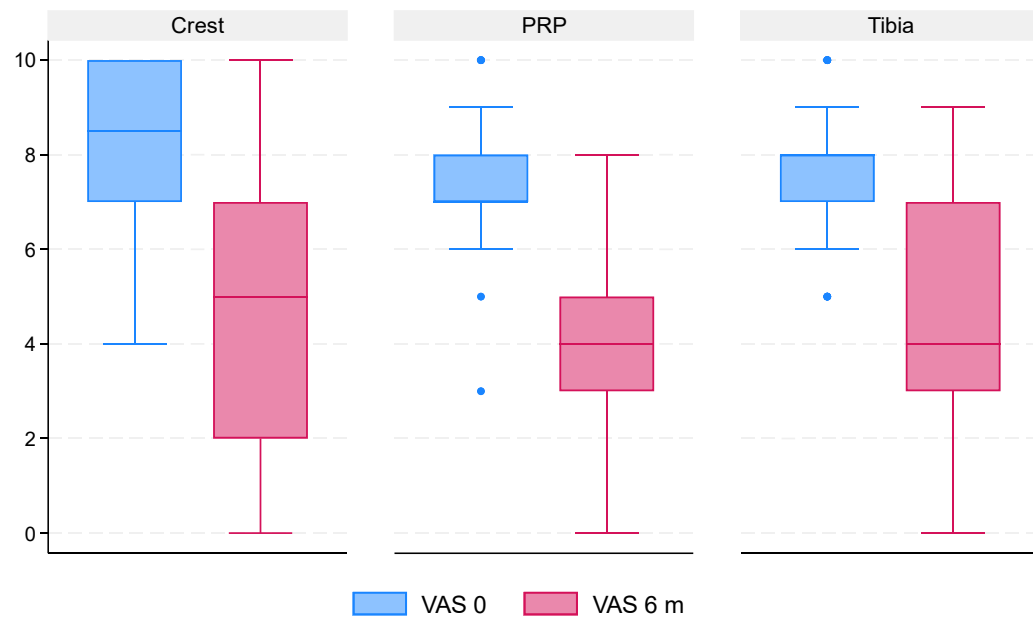

**b**

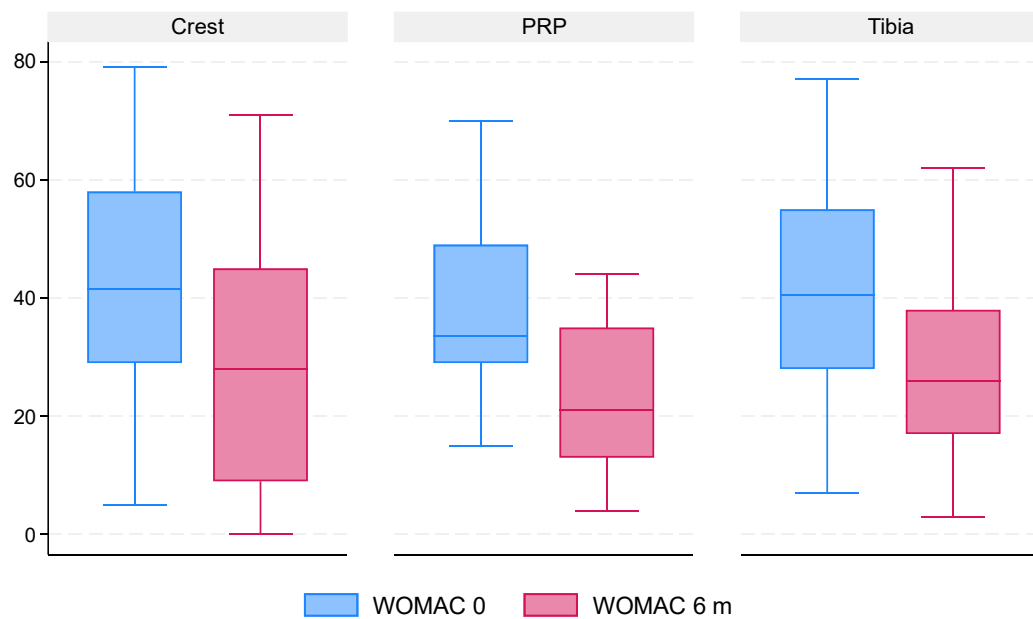

Supplement: Supplementary file 1 — Supplemental Fig Boxplots for VAS (a) and WOMAC (b) at T0 and T6 in the Crest, PRP, and Tibia Groups, respectively. [file JEO2-12-e70442-s003.pdf]
