# Supplementary material for: A randomised controlled trial confirms the non‐superiority of bone marrow aspirate (BMA) from the posterior iliac crest and proximal tibia compared to platelet rich plasma (PRP) in the treatment of knee osteoarthritis
Source: J Exp Orthop. 2025 Oct 9;12(4):e70442. doi: 10.1002/jeo2.70442 (PMC12509241; doi:10.1002/jeo2.70442)
Supplement: Supplementary file 2 — Table S1 new. [file JEO2-12-e70442-s002.pdf]

Table S1. Mean values and standard deviations (SDs) of 6-month changes in VAS and WOMAC scores across the three study groups.

**Variable**	**Crest**	**P**	**PRP**	**P**	**Tibia**	**P**
ΔVAS	-3.57 ± 3.15	<0.001	-3.77 ± 2.11	<0.001	-3.43 ± 2.9	<0.001
ΔWOMAC	-15.83 ± 19.48	<0.001	-15.70 ± 9.85	<0.001	-13.57 ± 18.85	<0.001
ΔWOMAC PAIN	-2.93 ± 4.25	0.001	-3.57 ± 2.39	<0.001	-2.73 ± 4.11	0.001
ΔWOMAC STIF	-1.67 ± 2.06	<0.001	-1.93 ± 1.23	<0.001	-1.03 ± 2.34	0.022
ΔWOMAC PF	-11.23 ± 14.2	<0.001	-10.23 ± 6.9	<0.001	-9.8 ± 13.4	<0.001

VAS, Visual Analogue Scale; WOMAC, Western Ontario and McMaster Universities Arthritis Index; PF, Physical Function.; Δ, 6-month change, calculated as post-treatment minus baseline value; P, p-value from the Wilcoxon matched-pairs test assessing the significance of differences between pre- and post-treatment within each arm.
