# Supplementary material for: A randomised controlled trial confirms the non‐superiority of bone marrow aspirate (BMA) from the posterior iliac crest and proximal tibia compared to platelet rich plasma (PRP) in the treatment of knee osteoarthritis
Source: J Exp Orthop. 2025 Oct 9;12(4):e70442. doi: 10.1002/jeo2.70442 (PMC12509241; doi:10.1002/jeo2.70442)
Supplement: Supplementary file 3 — Table S2. [file JEO2-12-e70442-s001.pdf]

Table 2. Summary of patient demographics, baseline clinical characteristics, bone marrow cellular and biochemical composition, and clinical outcome measures, presented separately for each of the three study groups.

	Study groups			
**Variable**	**Crest**	**PRP**	**Tibia**	**P**
N	30 (33.3%)	30 (33.3%)	30 (33.3%)	
**Demographic and baseline clinical characteristics**				
Sex female	17 (56.7%)	15 (50.0%)	13 (43.3%)	0.587
Age (years)	53.8 ± 7.9	51.3 ± 9.7	52.9 ± 8.8	0.559
BMI (Kg/m^2)	27.1 ± 3.2	26.8 ± 2.8	26.4 ± 2.8	0.704
Side R	19 (63.3%)	18 (60.0%)	14 (46.7%)	0.387
Kellgren-Lawrence Grading Scale				
I-II	18 (60.0%)	25 (83.3%)	16 (53.3%)	0.037
III-IV	12 (40.0%)	5 (16.7%)	14 (46.7%)	
HKA	-0.23 ± 1.87	-0.19 ± 2.16	0.23 ± 1.62	0.579
**Bone Marrow Cellular and Biochemical Composition**				
BM %	75.7 ± 16.3	–	40.2 ± 16.6	<0.001
MSCs (10^3 * %)	128.5 ± 111.7	–	2.1 ± 4	<0.001
MNCs (10^6/ml)	11.5 ± 6.3	–	5.7 ± 2.9	<0.001
Monocytes (10^6/ml)	2.8 ± 2.1	–	1.5 ± 1.2	0.007
Platelets (10^6/ml)	244.7 ± 76.2	–	178.3 ± 136.2	0.033
HCT %	39.3 ± 3.6	–	38.4 ± 8.3	0.620
**Clinical outcome measures**				
VAS at T0	8.43 ± 1.72	7.47 ± 1.53	7.63 ± 1.43	0.042
VAS at T6	4.87 ± 3.08	3.70 ± 2.07	4.20 ± 2.51	0.221
ΔVAS	-3.57 ± 3.15	-3.77 ± 2.11	-3.43 ± 2.90	0.895
WOMAC at T0	43.30 ± 20.74	38.37 ± 15.94	41.90 ± 18.80	0.573
WOMAC at T6	27.47 ± 19.43	22.67 ± 11.97	28.33 ± 16.52	0.352
ΔWOMAC	-15.83 ± 19.48	-15.70 ± 9.85	-13.57 ± 18.85	0.840
WOMAC Pain at T0	8.63 ± 4.15	8.07 ± 3.60	8.50 ± 3.65	0.834
WOMAC Pain at T6	5.70 ± 4.21	4.50 ± 2.61	5.77 ± 3.13	0.269
ΔWOMAC Pain	-2.93 ± 4.25	-3.57 ± 2.39	-2.73 ± 4.11	0.659
WOMAC Stiffness at T0	3.90 ± 2.09	3.90 ± 1.65	3.60 ± 2.22	0.799
WOMAC Stiffness at T6	2.23 ± 1.74	1.97 ± 1.38	2.57 ± 1.94	0.396
ΔWOMAC Stiffness	-1.67 ± 2.06	-1.93 ± 1.23	-1.03 ± 2.34	0.186
WOMAC ADL at T0	30.77 ± 15.29	26.40 ± 11.52	29.80 ± 13.94	0.433
WOMAC ADL at T6	19.53 ± 14.33	16.17 ± 8.77	20.00 ± 12.37	0.408
ΔWOMAC ADL	-11.23 ± 14.20	-10.23 ± 6.90	-9.80 ± 13.40	0.893

Means with standard deviations summarized continuous variables, while frequencies and percentages described categorical variables.

T0, baseline; T6, 6-month timepoint; Δ, absolute variation between T6 and T0 (T6 - T0); BMI, body mass index; HKA, hip-knee-ankle angle; BM%, percentage of bone marrow contribution; MSCs, mesenchymal stem cells; MNCs, mononuclear cells; HCT%, hematocrit percentage; VAS,

visual analog scale, WOMAC, Western Ontario and McMaster Universities osteoarthritis index; ADL, activities of daily living.
